# MausDB: An open source application for phenotype data and mouse colony management in large-scale mouse phenotyping projects

**DOI:** 10.1186/1471-2105-9-169

**Published:** 2008-03-26

**Authors:** Holger Maier, Christoph Lengger, Bruno Simic, Helmut Fuchs, Valérie Gailus-Durner, Martin Hrabé de Angelis

**Affiliations:** 1 Helmholtz Zentrum München – German Research Center for Environmental Health, Institute of Experimental Genetics (IEG), Ingolstädter Landstr. 1, D-85764 Neuherberg, Germany; 2 ExpertMind, Neuried, Germany; 3 Lehrstuhl für Experimentelle Genetik, Technische Universität München, D-85350 Freising-Weihenstephan, Germany

## Abstract

**Background:**

Large-scale, comprehensive and standardized high-throughput mouse phenotyping has been established as a tool of functional genome research by the German Mouse Clinic and others. In all these projects, vast amounts of data are continuously generated and need to be stored, prepared for data-mining procedures and eventually be made publicly available. Thus, central storage and integrated management of mouse phenotype data, genotype data, metadata and linked external data are highly important. Requirements most probably depend on the individual mouse housing unit or project and the demand for either very specific individual database solutions or very flexible solutions that can be easily adapted to local demands. Not every group has the resources and/or the know-how to develop software for this purpose. A database application has been developed for the German Mouse Clinic in order to meet all requirements mentioned above.

**Results:**

We present MausDB, the German Mouse Clinic web-based database application that integrates standard mouse colony management, phenotyping workflow scheduling features and mouse phenotyping result data management. It links mouse phenotype data with genotype data, metadata and external data such as public web databases, which is a prerequisite for comprehensive data analysis and mining. We describe how this can be achieved with a lean and user-friendly system built on open standards.

**Conclusion:**

MausDB is suited for large-scale, high-throughput phenotyping facilities but can also be used exclusively for mouse colony management within smaller units or projects. The system is successfully used as the primary mouse and data management tool of the German Mouse Clinic and other mouse facilities. We offer MausDB to the scientific community as open source software to provide a system for storage of data from functional genomics projects in a well-structured, easily accessible form.

## Background

The concept of standardized, high-throughput and comprehensive screening of mice has proven to be successful for identifying new phenotypes in mutant mouse lines by the German Mouse Clinic (GMC) [1-7] and others [8,9].

In the GMC, experts from various fields of mouse behavior, physiology, morphology, metabolism and pathology work side-by-side in one building in 14 individual modules (allergy, behavior, cardiovascular system, clinical chemistry, dysmorphology, energy metabolism, eye development and vision, immunology, lung function, molecular phenotyping, neurology, nociception, pathology and steroid metabolism) in close collaboration with clinicians and veterinarians [2].

Mouse mutants and their littermate controls pass through the different modules of the GMC in multi-parallel phenotyping pipelines following a standardized workflow. In the course of the high-throughput primary screen, up to 320 parameters per mouse line are measured, and these findings may be supplemented by results from secondary and tertiary screening assays. In addition, individual modules may conduct independent projects and/or more intensive phenotyping procedures not included in the primary screen.

As a consequence, data integration is a major issue in the GMC, and appropriate bioinformatics support as well as well-defined data structures and processes are required. Data should preferably be stored in a central database to ease the identification of genotype-specific phenotypes or correlations between parameters measured in different modules and to perform cross-line comparisons. Central data management is crucial for integration of measured phenotype data with metadata (*e.g*. standard operating procedures (SOPs), experimental and housing conditions, etc.) and external data (*e.g*. linking of mouse genotype data with public databases). As an example for the integration of local data with external data, locally defined gene loci can be cross-linked to external information by attaching URLs pointing to public resources such as MGI or Ensembl. This feature reduces redundant information retrieval on the user side, facilitates discussion of phenotyping results and can be additionally used to cross-link databases for data mining purposes. Thus, downstream data analysis and data mining tools can access a central data resource rather than multiple distributed spreadsheet files. Central data management also facilitates quality control, data curation and backup as well as data exchange, *e.g*. within the cross-European phenotyping effort EUMODIC (European Mouse Disease Clinic) [10].

In addition to the scientific and phenotyping data-related aspects, an integrated mouse information and management system must also support mouse husbandry and mouse house management. In the GMC, mouse lines from all over the world are imported for primary screen phenotyping and bred for secondary or tertiary phenotyping or for individual research projects. In order to centrally manage shared resources such as rooms, racks, cages and personnel, all animals need to be managed and tracked by the same system.

Common to all mice in the GMC and other mouse facilities at the Helmholtz Zentrum München is the need for documentation of all aspects of a mouse and its life, including sex, genotype, date of birth, origin (import or weaning), date and reason of death, kind of genetic modification and use in experiments that are subject to authorization. Some of these data have to be reported to local authorities on a regular basis.

Several mouse database systems have been developed and published in the course of other large phenotype screening projects during the last years [11-14], and a couple of additional mouse database systems are commercially available. Despite the existence of these high-quality systems, we opted to develop a system for the needs of the GMC rather than to adapt third-party products to our requirements or to adapt our requirements to the features of third-party products. Therefore, we developed MausDB as a tool that meets all demands of the GMC mentioned above.

## Implementation

MausDB is set up as a typical LAMP system. In this context, the acronym describes the combined use of Linux as the operating system, Apache as the web server, MySQL as the relational database management system and Perl as the scripting language.

Since ease of installation and administration were major issues when setting up MausDB on our servers, we decided to use the Ubuntu Linux distribution (version 6.06 LTS). In our hands, the whole system, including all necessary packages for MausDB and MausDB-specific program files and databases, can be installed on a blank computer starting from a Ubuntu Linux 6.06 LTS live CD in less than 1 hour.

The hardware requirements of MausDB on the server side are moderate. Although our production server for the GMC (60+ total users, ~15 concurrent users) is a dual processor system (Intel Xeon, 3.06 GHz) with 4 GB RAM, MausDB also runs smoothly on a simple desktop computer with a single 2 GHz CPU and 1 GB RAM with the same number of users.

## Results and Discussion

### Overview

MausDB is a web-based application fully built on free standards (Linux operating system, Apache Web server, MySQL database, Perl as programming language). Non-redundant storage of data in a central database ensures integrity and consistency of data. Using a central database with an adequate backup strategy and administration also improves sustainability of scientific data and helps prevent data loss. Multiple users can simultaneously access the database *via *a web browser from their individual client computers no matter which operating system they use.

Although MausDB was primarily developed for the needs of the GMC, it has also turned out to be a valuable tool for other mouse facilities at the Helmholtz Zentrum München due to its flexible and general-purpose design.

As of January 2008, data of around 90,000 mice from four large mouse facilities at the Helmholtz Zentrum München – German Research Center for Environmental Health, including the GMC, are managed using MausDB.

### Basic concepts

Our objectives during development of MausDB were primarily to meet the functional requirements described above, but acceptance of the new system by its prospective users was also of prime importance. Usefulness and usability are the main essential issues with respect to user acceptance, especially in a quite heterogeneous environment. Usability is closely linked with convenience and ease of use, so we put much effort into development of a user-friendly interface.

### Ease of use

Intuitive use helps to reduce errors that are produced by user interaction, and ease of use also helps minimize the effort for user training. We applied user interaction concepts that most everyone is familiar with from other World Wide Web contexts. For example, we implemented a mouse "cart", which can be used to first collect a set of mice and then apply a common procedure (*e.g*. mating, genotyping, culling or moving to another cage) to the selected mice; as most Internet shops use a virtual "shopping cart", no specific training is needed to instruct users how to do the same thing with mice.

Since we identified abbreviations and cryptic language as a major barrier to usability, we use clear and non-ambiguous English language in the user interface and avoid the use of abbreviations as much as possible.

### Flexibility with only a few strict rules

The GMC has a strict workflow for mice subjected to primary screening. On the other hand, many mice are imported or bred for secondary screening research projects by the individual scientists from different screening modules. This is reflected by a large number of – sometimes mutually contradictory – user requirements for handling even standard operations such as mating, weaning or mouse movement.

To cope with all these specifications, we implemented only few basic rules. Strict rules are not necessary in all cases: there is no need, for example, to strictly prevent mice with the same ear marks from being in the same cage, as there might be additional attributes that help to distinguish mice. In the same example, it also makes no sense to apply strict rules on the database level when the physical movement has already been performed.

Thus, MausDB follows the convention to only generate a warning in such error-prone situations and let the user decide whether to ignore the warning or not. Therefore, MausDB users are more in charge of the correctness of their input than users of other systems that may apply stricter or more complex rules. On the other hand, this flexibility provides the opportunity to use MausDB in a quite heterogeneous environment without the need to define and administer project-specific rules. In addition, the complexity of the system can be kept very low, as every rule might create new dependencies.

### Lean administration

To minimize the need for intervention by database or system administrators, corrections of false entries that need to be done regularly (*e.g*. update sex) or have little or no side effects (*e.g*. update ear marks) can be done by the users on their own without having to contact an administrator.

Some tools (*e.g*. check database integrity, database statistics) and frequently needed administrative task dialogs (*e.g*. adding new users, setting up new rooms and racks, defining new mouse lines) are integrated into the MausDB web user interface but are restricted to users with administrative privileges. No SQL experience is needed for this kind of daily routine administration.

In the current version of MausDB, some complex or infrequent operations require inserting or altering data on the database level, where basic SQL experience is necessary.

Customizing the user interface or adding new features is straightforward but requires advanced Perl and SQL skills.

### MausDB features and capabilities

#### Phenotyping workflow management

In the GMC, every screening module offers the measurement of different parameters, which are grouped within standardized assays or so-called parameter sets. For example, the neurology module screens mice following a modified SHIRPA (SmithKline Beecham Pharmaceuticals; Harwell, MRC Mouse Genome Centre and Mammalian Genetics Unit; Imperial College School of Medicine at St. Mary's; Royal London Hospital, Phenotype Assessment) protocol [15] that includes 23 individual parameters. The parameters and assays have to be defined on the database level using SQL commands, as there is currently no graphical user interface for this purpose. Defining new parameters or assays does not require modification of the source code; everything can be easily configured on the database level.

Mutant mouse lines subjected to primary screening enter the GMC in general at the age of 5 weeks and pass the different screening modules in a strictly defined order, the so-called primary workflow [2].

**Figure 1:**
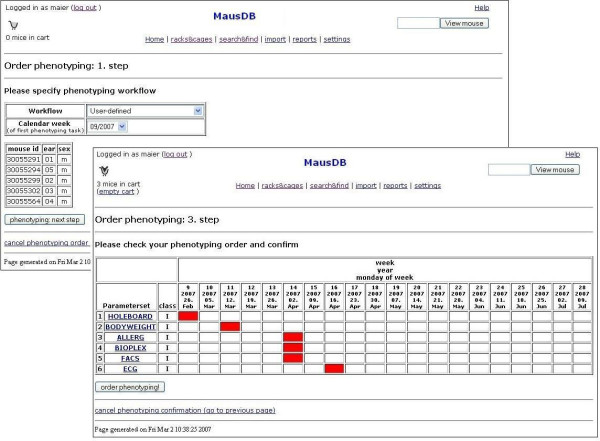
**Workflow management**. Upper left: a specific or custom workflow (a set of phenotyping assays in a distinct order) can be scheduled for a selection of mice. Lower right: a detailed schedule table (rows: phenotyping assays; columns: calendar weeks) is displayed as a final preview. Red boxes denote the calendar week in which the given assay is to be performed.

Specific work lists contain individual mice and the assays that are scheduled for a certain week. Once defined by core facility members upon mouse import, these work lists are displayed automatically to technicians and scientists in their respective modules when logging in to MausDB. In the GMC, this automatic scheduling and reminder system is used to inform technicians and scientists when specific mice should be analyzed. As many mutant mouse lines are subjected to primary phenotyping in parallel within shifting pipelines, workflow scheduling management support is an essential feature of MausDB for the GMC. In addition to scheduling of routine primary screening, work list management can be customized and used independently in every module to ease project management (Figure 1).

**Figure 2:**
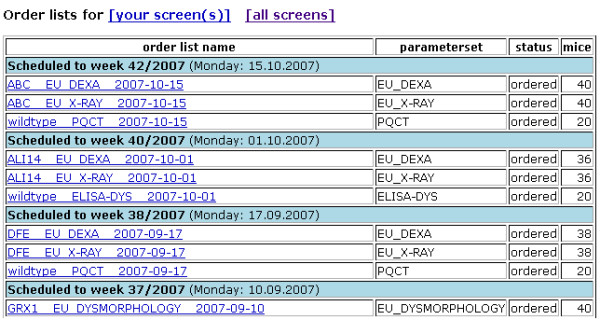
**Phenotyping ordering system: reminder list**. Upon login, a reminder list of ordered phenotyping tasks is displayed to every user. The list is sorted by calendar week and only contains phenotyping orders to be performed in the module to which a user is assigned. The name of the phenotyping task ("order list", left column) is composed of the mouse line, the assay to be measured and the due date. The rightmost column specifies the number of mice to be phenotyped in this order, whereas the 'parameterset' column denotes the assay to be performed. The order list link in the left column is clickable and leads to a more detailed view of the phenotyping order.

**Figure 3:**
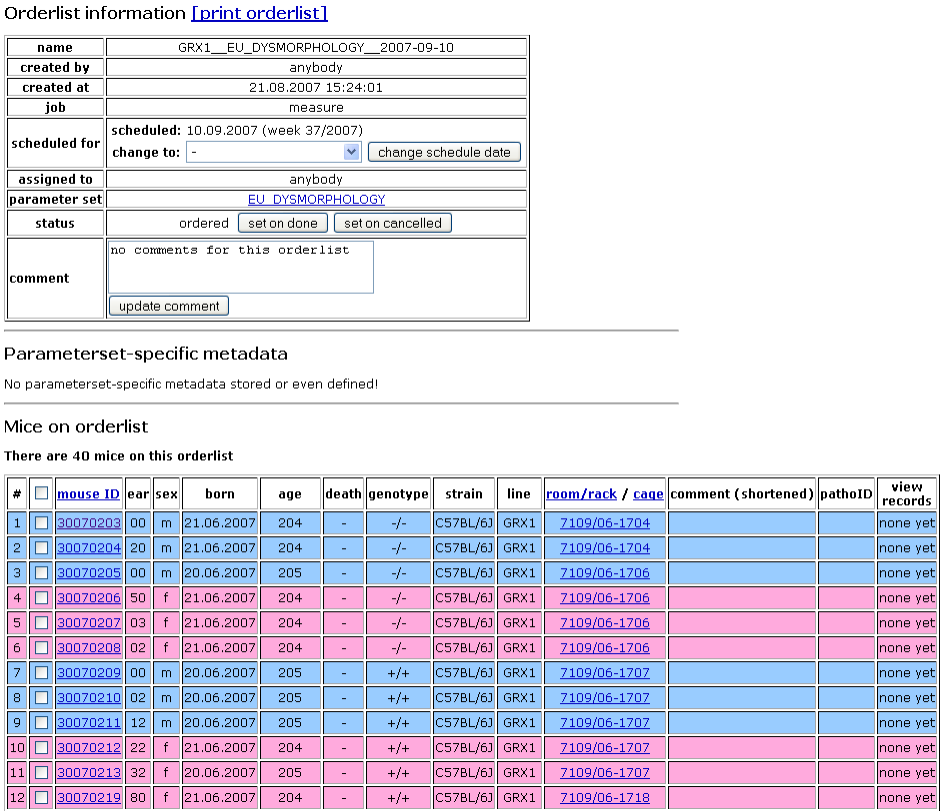
**Phenotyping ordering system: phenotyping order list**. A detailed view of every order list is available by following the link in the 'order list name' column of the reminder list (cf. Figure 2). The top table contains information on the order list, *e.g*. creation data, schedule date and a link to the parameter set (*i.e*. assay) to be measured. The scheduled date as well as the phenotyping order status can be modified using the respective controls. The bottom table is a list of all mice to be analyzed in the current phenotyping order along with important information,*e.g*. age, sex, genotype, line and current location.

Work lists are assigned and automatically displayed to all users belonging to a certain project (Figures 2, 3). The work lists are ordered by calendar week but cannot be re-ordered or prioritized by the users, as this is not required in the GMC. In this respect, the version of MausDB described here provides basic but not extensive task and project management tools supporting a central facility management team.

#### Phenotype data management

In general, spreadsheet files are produced directly by, for example, a blood analyzer or grip strength meter. However, for specific needs, spreadsheet files can be generated manually by the screeners or are generated *via *export from module-specific databases. Uploading of phenotyping results is straightforward and works by simply uploading the appropriate spreadsheet file *via *the web interface. This approach is quite universal and can be used by almost any institution by configuring the settings on the database level, without changing the source code.

During the uploading procedure, the full path and file name of the spreadsheet file as well as the sheet name containing the results are requested interactively. The result sheets have to have a standardized, assay-specific matrix format: the results from one mouse are arranged in one row, with the columns representing mouse ID, date of measurement and the different phenotyping parameters of the assay. The uploading procedure includes checking of data type (float, integer, text, Boolean) and plausibility checking of parametric results, mouse IDs and dates (to some extent using regular expressions). The column header names and the column position used in the result file are compared with expected values stored in the database for each assay. Undefined, additional columns are ignored. A color-coded warning is displayed for every spreadsheet field with a missing value. Critical errors such as invalid or missing mouse ID and date, missing or displaced columns or wrong data type cause an abortion of the uploading procedure. Bounds and ranges for plausibility checks can be defined for every parameter in the database, and these additional checks can be plugged easily into the uploading procedure. In the last step of the uploading procedure, a final visual inspection of the result matrix has to be performed by the user before the results are inserted into the database.

**Figure 4:**
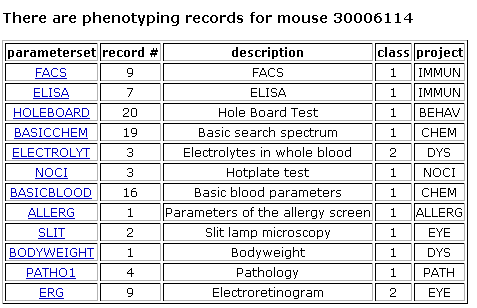
**Phenotyping records overview for a mouse**. For every mouse, the phenotyping records overview summarizes available phenotyping data from every parameter set (*i.e*. assay). In the left column, the short names of the corresponding phenotyping parameter sets are specified, while the column 'description' explains the parameter sets in more detail. The 'record #' column shows the number of phenotyping records for every parameter set. The 'class' column names the classification of the parameter set (1: primary screen, 2: secondary screen). The 'project' column specifies the assigned project (*i.e*. phenotyping module) for a parameter set.

**Figure 5:**
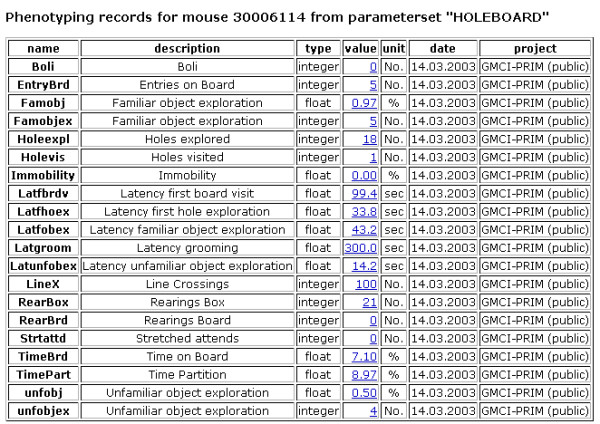
**Phenotype data for a mouse from one parameter set (assay)**. The complete list of phenotyping data from a particular parameter set (assay) is accessible by following the link in the first column of the phenotyping record overview (cf. Figure 4). The table shows (from left to right) the short names and the descriptions of the parameters, their data type (integer, float, Boolean, or text), the individually measured values and their units, the date of measurement and the project assignment, which is relevant for access permission.

**Figure 6:**
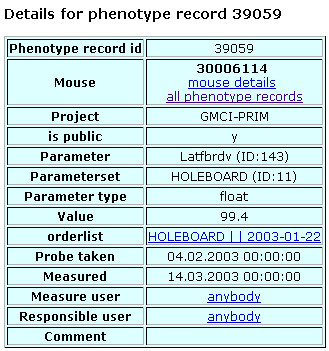
**Detailed view of a single phenotype record**. For every phenotyping record, a more detailed view is available by following the link in the 'value' column of the phenotyping data list (cf. Figure 5). This view additionally offers links to the corresponding order list and the parameter set description and shows the accessibility of the result ('is public'). In the 'probe taken' and 'measured' fields, date and time of sample taking and measurement, respectively, are shown. If the time is not given by the user, 00:00:00 is used instead. 'Measure user' names the screener who performed the measurement, while 'Responsible user' names the scientist who checked the results for validity before they were uploaded.

**Figure 7:**
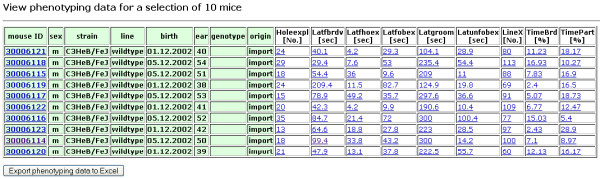
**Table view of phenotype data for a set of mice**. For a selection of mice from the cart, a customizable phenotype data table can be generated for selected parameters. Next to individual mouse metadata (grey columns), phenotype data values (white columns) are displayed. The table can be readily exported into a spreadsheet file, which can be downloaded from the MausDB server to the client computer by clicking the 'Export phenotyping data to Excel' button.

Once uploaded into MausDB, phenotyping data for individual mice or groups of mice can easily be accessed and exported (Figures 4, 5, 6, 7). Assay-specific metadata, *i.e*. SOPs or descriptions of experimental and housing conditions, can be attached to the phenotyping data upon upload.

In addition to the uploading of pre-defined parametric data, any file (for example, spreadsheet files, image files or expression chip analysis files) can be uploaded and permanently attached to a mouse or a group of mice.

MausDB does not currently use any ontologies to store phenotype data, but this will be a feature of future versions. In addition, the use of controlled vocabularies for the collection of phenotype data will be implemented.

#### Mouse management and husbandry

##### Individual IDs

Standard animal management tasks are probably very similar in most mouse facilities. In MausDB every mouse has its own, unique ID. In terms of quality and good practice, this property of MausDB is essential for its use in the GMC.

**Figure 8:**
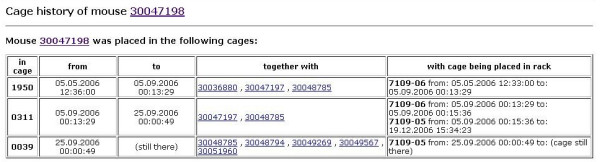
**Cage history of a mouse**. For any given mouse, the full history of cage placements, including time spent in the respective cage and cage mates during this time period, can be accessed. As cages keep their ID when moved between rooms and racks, the rightmost column informs about cage placement in rack(s).

Straightforward dialogues allow import, mating, embryo transfer, weaning, culling and genotyping of mice. Mice can be moved between cages, racks and rooms, with full preservation of location history; as a result, the full cage mate history can be queried for any mouse, which can become a quite important feature in the context of infections and sanitary monitoring [16] (Figure 8).

##### Grouping of mice using the "cart"

Regardless of where they are actually located, mice can be grouped by virtually putting them in the so-called "cart". Carts are attached to the browser session, allowing temporary grouping of mice, but they can also be stored permanently for public or private use and reloaded later on. This feature of the cart system is very useful in the course of the primary screening workflow: mouse cohorts stay in the GMC for 14 weeks, during which they are sequentially moved to 11 independent screening modules. During this time, the mice may be put into other cages and examined in different assays, but they always stay grouped together in their original "cart".

**Figure 9:**
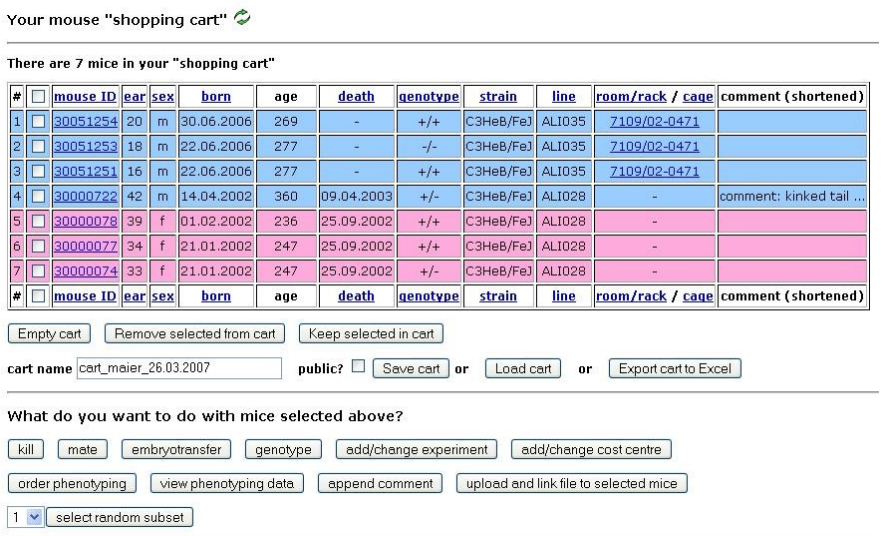
**Mouse "shopping cart"**. Mice can be placed together in a cart irrespective of their real rack or cage location; the cart can also contain dead mice. Table: For each mouse in the cart, the basic data (ID, ear mark, sex, date of birth, date of death, genotype, strain, line, room/rack/cage and comment) is displayed in a row. The background color of the mouse rows is sex-specific (blue: male; pink: female). Information about current location (room, rack and cage) is given for living mice. Individual mice can be selected *via *checkbox (second column) for further operations. The table can be sorted by clicking on the respective underlined header column. The cart can be emptied all at once, or individually selected mice can be removed or kept in the cart. Carts can be saved permanently with a custom name and reloaded later. Table information can be exported to Excel or another spreadsheet application. Bottom: Pressing one of the "buttons" at the bottom part of the cart view applies the respective operation to selected mice.

In general, the cart allows grouping of mice for any purpose. Data for mice in a specific cart can also be easily exported in spreadsheet format for further processing (Figure 9).

##### Search & find functions

**Figure 10:**
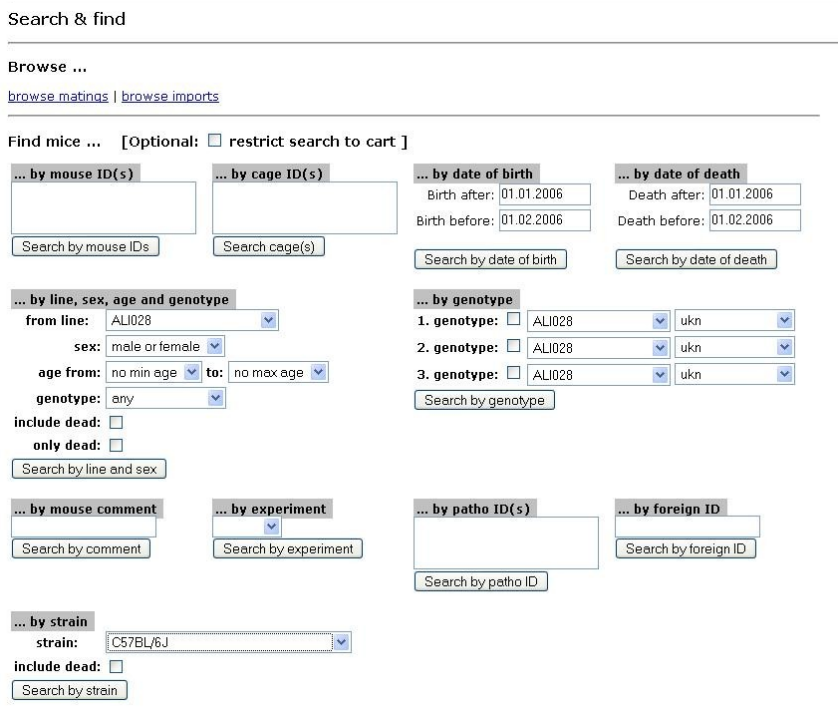
**Search & find mask of MausDB**. Mice can be searched for using different attributes as starting information, *e.g*. mouse ID, cage ID, date of birth or date of death, line, genotype or part of the mouse comment. As an option, searches can be restricted to mice currently in the cart, which allows complex search operations to be performed.

**Figure 11:**
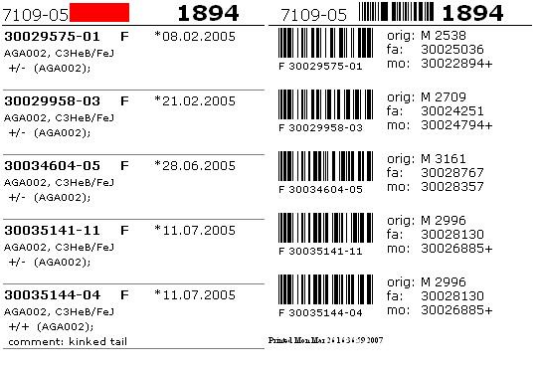
**Cage card example**. Front side (left part): the top row contains room (7109), rack (05) and cage (1894) information. The bar color can be freely assigned by the user and may visually encode the line or any other information in a user context. Mouse-specific rows contain the mouse ID together with ear mark, sex and date of birth (first row); line and strain/background (second row); genotype data (third row) and mouse comment (fourth row). Back side (right part): the top row contains room, rack and cage number as well as a bar code representation (Code39) of the cage number. Mouse-specific columns contain bar code representations (ITF) of the mouse ID as well as the mouse ID as text together with sex and ear mark in the left column. In the right column, the origin (orig) of a mouse is denoted as 'M' (mating) or 'I' (import), followed by the respective mating or import ID. When originating from a mating, father (fa) and mother (mo) IDs are printed. The mother ID is followed by '+' when more than one mother is assigned.

**Figure 12:**
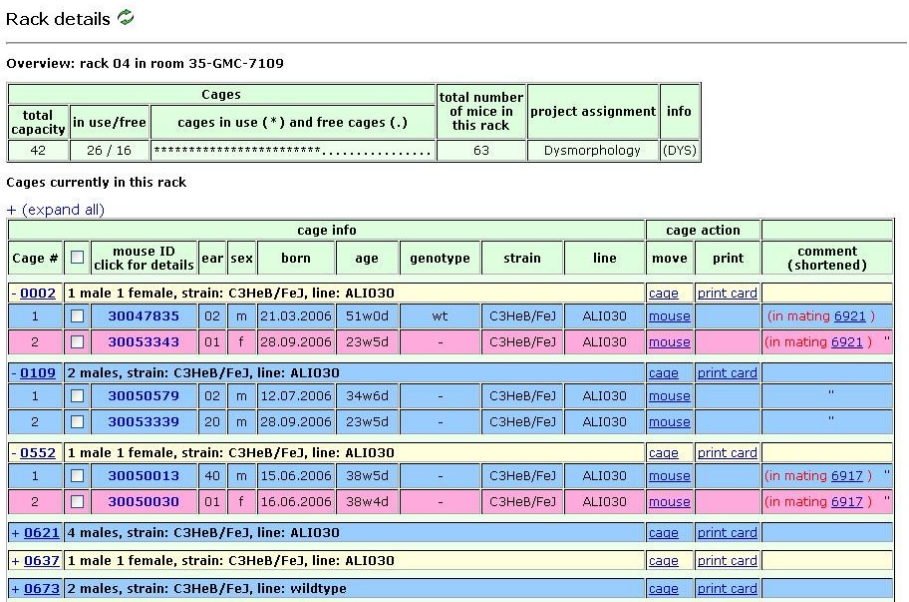
**Rack view**. Top table: the rack overview contains general information about rack capacity, cages in use, free cage slots and total mice in the rack. Bottom table: this table lists every cage currently in the rack. Cages can be viewed in expanded mode (cages 0002, 0109 and 0522 in this example) or in condensed mode, which is the default. In expanded mode, every mouse together with basic data (ID, ear mark, sex, date of birth, genotype, strain, line and comment) is displayed in a row. The background color of the mouse rows is sex-specific (blue: male; pink: female). Individual mice can be selected from all expanded cages *via *checkbox (second column) or moved to another cage (third column from the right). In condensed mode, only cage summary information (number of mice per sex, strain and line) is displayed. The whole cage can be moved to another rack (third column from the right), or a cage card can be printed. The background color of the cage rows is sex-specific (blue: male; pink: female; yellow: mixed cages).

Extensive search & find functions (Figure 10) as well as printing of cage cards with barcodes (Figure 11) allow fast tracking of mice. Browse functions include room and rack view (Figure 12), cage view and mouse detail view as well as browsing lists (and detailed views) of all imports and matings.

Searches can be restricted to mice in the session cart. Thus, by combining the use of search & find functions and the cart, complex search operations can be performed.

##### Multiple genotypes

For each mouse, MausDB can manage multiple mutant alleles and their respective genotypes, which can be assigned either individually or for a selection of mice *via *the cart.

##### Genealogy

**Figure 13:**
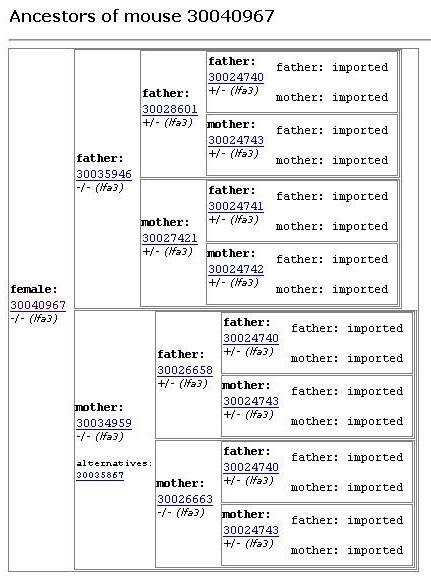
**Ancestor table**. A pseudo-graphic, table-based view shows the ancestors of a given mouse (leftmost column). For every mouse, father and mother are displayed together with their ID and genotype.

Parental relationships are fully defined and stored for every mouse in the database. Thus, MausDB allows easy identification of parents of a given mouse or offspring of a given breeding pair. For any mouse, an ancestor table spanning five generations and including genotypes can be displayed (Figure 13).

### Intended use

MausDB is designed to cope with thousands to tens of thousands of concurrently living mice in large mouse facilities. As an integrated system, it can be used for managing mouse breeding and phenotype data as well as scheduling screening workflow in such phenotyping centers.

Although MausDB is designed for rather large projects, it can still be used for small-scale mouse stock breeding with only a few racks. Using the cart and the phenotyping order management tools, MausDB can be used in fully managed units, where a central management team coordinates tasks to be performed by technicians and animal keepers, though these management tools might need further improvement. On the other hand, MausDB can also be used in decentralized mouse facilities, where different independent groups operate on their own without being directed by a central management team.

### Benefits of MausDB

MausDB is freely available open source software and thereby can help to reduce costs. Download, use and adaptation or further development of MausDB is not only allowed, but encouraged. From our experience, MausDB also helps to reduce the amount of time spent with mouse colony and data management because information is centrally stored and accessible for concurrent read and write access by many users.

Projects sharing mouse space in a central facility can profit from sharing hardware (computers and cage card printers) and personnel trained in using a common mouse colony management system.

In comparison to distributed spreadsheet files or paper-based laboratory journals, the use of MausDB helps to improve overall data quality, as changes are made to a central database and are checked for plausibility.

Storage of structured data in a central relational database is also a prerequisite for integrating specific phenotyping data with data from public databases. As a consequence, the application of data mining methods to phenotyping data is significantly facilitated.

### Planned future developments

We intend to implement new features for the documentation of treatments on the level of individual mice, such as exposure to environmental challenges or medication. In addition, integration of tools for basic statistical analysis, data visualization and data mining is planned. Integration of ontologies and controlled vocabularies for the collection of phenotype data will also be implemented in future versions of MausDB.

## Conclusion

We have developed an integrated phenotyping workflow, data and mouse management system named MausDB that can be used by mouse facilities ranging from large-scale, high-throughput phenotype screening facilities to small mouse stock breeding units. MausDB centrally stores and integrates phenotype data with mouse husbandry data (*e.g*. line, genotype) and other metadata on the level of individual mice, allowing access by data analysis and data mining tools. The MausDB web interface is very intuitive and user-friendly, which reduces the need for user training to a minimum. Due to its lean and open design, it can be easily installed and adapted for custom purposes. We offer MausDB to the scientific community as open source software under the terms of the GNU General Public License (GPL).

## Availability and requirements

**Project name: **MausDB

**Project home page: ** (section "downloads")

**Operating system: **platform-independent

**Programming language: **Perl

**Other requirements: **server: Apache 1.3 or above, MySQL 4.23 or above; client: Mozilla Firefox (Windows XP, Linux and Mac OS X) or Safari (on Mac OS X). JavaScript and cookies need to be activated for full functionality.

**License: **GNU GPL

**Any restrictions for use by non-academics: **none

## Authors' contributions

HM conceptually designed and implemented the MausDB user interface and the underlying database and drafted the manuscript. CL made substantial contributions to the conception and design of MausDB, provided the data model of the German Mouse Clinic and helped to draft the manuscript. BS developed methods for data acquisition, data validation and migration of existing data from a previously used database. HF and VGD helped to draft the manuscript, revised it critically and participated in coordination of the development process. MHdA revised the manuscript critically and gave final approval of publication. All authors read and approved the final manuscript.
